# The Magnificent Century of Cardiothoracic Surgery Part 8: Reviving the Dead

**DOI:** 10.4103/1995-705X.73228

**Published:** 2010

**Authors:** Amer Chaikhouni

**Affiliations:** Department of Cardiothoracic Surgery, Hamad General Hospital, Doha Qatar

**“Coming together is a beginning Keeping together is progress. Working together is success.”**

*Henry Ford*

The story of developing cardiac pacemakers is a story of team work. Very much like many great medical inventions developed in the Twentieth Century.

During the Eighteenth Century, Scientists were fascinated by electricity and many experiments were conducted to study this new exciting field. In 1775, the Danish physicist Nickolev Abildgaard placed electrodes on the sides of a hen’s head and applied an electric discharge, which made the hen fall dead. When the electrodes were applied over the hen’s chest, it staggered onto its feet and walked away! It was like a magical revival of the dead. Great minds, such as Luigi Galvani (1737 – 1798) and Alessandro Volta (1745 – 1827) in Italy, Charles-Augustine de Coulomb (1736 – 1806) and André-Marie Ampère (1775 – 1836) in France, George Ohm (1789 – 1854) in Germany, and Michael Faraday (1791 – 1867) in England, were conducting important experiments, which established the basic understanding of electricity. Doctors were also involved in such research very early.

In 1786, Luigi Galvani (1737 – 1798) an Italian professor of medicine, found that when the leg of a dead frog was touched by a metal knife, the leg twitched violently. Galvani thought that the muscles of the frog contained electricity, and he wrote about ‘animal electricity’. By 1792, another Italian scientist, Alessandro Volta, realized that the main factors in Galvani’s discovery were the two different metals, the steel knife and the tin plate upon which the frog was lying. Volta showed that when moisture comes between two different metals, electricity is generated. This led him to invent the first electric battery, the voltaic pile. In this way, a new kind of electricity was discovered, electricity that flowed steadily like a current, instead of discharging itself in a single spark. The instrument for measuring and recording electricity is named ‘Galvanometer’ after Galvani, and ECG is essentially a sensitive galvanometer. The unit of electrical potential, the Volt, is named after Volta.

John Hunter (1728 – 1793), a Scottish surgeon who is one of the most distinguished pioneers of experimental surgery, in 1776, recommended that electric stimulation should be tried in the resuscitation of drowning victims. In London, Charles Kite attempted that in 1788. In “*An essay upon the recovery of the apparently dead*” he reported the use of an electrostatic generator (Layden jar) in the resuscitation of a three-year-old girl who was supposed to be dead after falling out of a window. In his own words he described the event: “*with the consent of the parents, very humanely tried the effects of electricity. Twenty minutes had at least elapsed before I could apply the shock, which I gave to various parts of the body without any apparent success; but at length, on transmitting a few shocks through the thorax, I perceived a small pulsation; soon after that the child began to breathe, though with great difficulty. In about 10 minutes she vomited. A kind of stupor she remained for some days, but the child was restored to perfect health and spirit in about a week.*” He went on to present his sound scientific vision in declaring that: “…*electricity is the most powerful stimulus we can apply…if it is able so powerfully to excite the action of the external muscles, it will be capable of reproducing the motion of the heart which is infinitely more irritable, and by that means accomplish our great desideratum, the renewal of the circulation.”*

## THE FRANKENSTEIN OF MARY SHELLEY; FICTIONAL REVIVING OF THE DEAD

Tambora’s 1815 outburst in Indonesia was the largest volcanic eruption in recorded history. The explosion was heard more than 2,000 km away. The death toll was at least 71,000 people (the most deadly eruption in recorded history). The eruption created global climate changes that included the phenomenon known as ‘volcanic winter’: 1816 became known as the ‘Year without summer’ because of the cooling effect on North American and European weather. Agriculturai crops failed and livestock died in much of the Northern Hemisphere, resulting in the worst famine of the Nineteenth century. During that dreadful summer of 1816, Mary Shelley, aged 18, and her lover (and later husband) Percy Shelley, visited Lord Byron in Switzerland. Among other subjects, conversations turned to galvanism and the feasibility of returning a corpse or assembled body parts to life, and to the experiments of the eighteenth-century natural philosopher and poet Erasmus Darwin, who was said to have revived dead matter. Inspired by this situation, the young writer Mary Shelley came up with the horrific science fiction novel ‘Frankenstein,’ which was published in 1818. The novel told the story of Victor Frankenstein, the bioscientist who managed to assemble parts of dead human bodies and ‘bring them back to life’ by lightning, to create the famous nameless monster that was mistakenly named later after the name of the ‘mad’ scientist… Frankenstein!

## EARLY SHOCKS

In 1899, Jean-Louis Prévost (1838 – 1927) and Frederic Batelli in Geneva demonstrated that electrical currents could cause ventricular fibrillation, which could often be reversed by another powerful stimulus of either alternating or direct current to the canine heart, thereby inventing the first electrical defibrillator. John A. McWilliam (1857 – 1937) of Scotland published a series of articles, between 1887 and 1899, on cardiac electrophysiology in mammals, in which he identified an area of the sino-atrial node, studied the development and progression of ventricular fibrillation, postulated that sudden death in humans could be the result of ventricular fibrillation, and proved that it was possible to evoke cardiac contractions by electric stimuli applied directly to the heart. Louise Robinovitch, a pioneer woman in American medicine, a graduate of the Women’s Medical College of Pennsylvania in 1889, continued her studies and research in France in the 1900s. She discovered that pulsed electrical stimuli could be used for cardiac resuscitation. She designed equipment, which was the forerunner of the automatic defibrillator. She published a series of reports from 1906 through 1909, designed the first portable electrical resuscitative apparatus for ambulances and almost came up with an external pacemaker device. Interestingly, the significance of these sporadic experimental and clinical reports was not well appreciated at the time because of the lack of understanding of ventricular fibrillation. Electric defibrillation was considered impractical and applications of either cardiac pacing or electrical defibrillation were delayed.

## EARLY DEFIBRILLATION RESEARCH IN USSR

In 1938 – 1939, impressive research on electrophysiology was performed by the pioneer Naum Lazarevich Gurvich (1905 – 1981), in Moscow. He investigated the effects of alternating (AC) and direct (DC) electric currents on the heart. His study was published in English in 1945. He demonstrated the advantages of DC over AC shock, and even proposed the biphasic waveform defibrillation at that early time. His defibrillator was used clinically in cardiac operations in 1952, in USSR. His biphasic waveform defibrillation had to wait another 50 years before it was clinically applied in external and implantable defibrillators. Gurvich was awarded the National Award of USSR in 1970.

## THE FIRST CLINICAL DEFIBRILLATION IN HUMANS; ACTUAL REVIVING OF THE DEAD?

It was not until the efforts of William Kounwenhowen (1932), Naum Gurvich (1939), and Carl J. Wiggers and Claude Beck (1947) that electrical defibrillation became widely used. A boy, 14 years of age, was operated by the American cardiac surgeon Claude Schaeffer Beck (1894 – 1971), for repair of severe funnel chest. During the closure of the chest incision, the pulse suddenly stopped and blood pressure could not be recorded. The patient was apparently dead. The wound was reopened and cardiac massage was initiated. Cardiac massage was continuously given for 35 minutes, at the end of which the electrocardiogram was characteristic of ventricular fibrillation. A first electric shock applied directly to the heart was unsuccessful. Procaine hydrochloride was injected into the right atrium, the heart massaged and a second series of shocks given. The heart then was in standstill. However, feeble, regular, and fairly rapid cardiac contractions appeared. Cardiac massage was continued for five more minutes, at which time it was obvious that the contractions became coordinated and fairly vigorous, although still fast. The electrocardiogram at this time revealed supraventricular tachycardia at a rate of 175. The heart gradually increased in vigor and brachial blood pressure was 50. This was a report of the first case, published in 1947, with complete recovery after prolonged ventricular fibrillation. Beck suggested that operating rooms should be equipped with electric shock devices to treat cases of sudden ventricular fibrillation, and that personnel should be trained in the method. He also recommended that speed and precision in the technique were important!

These early defibrillators were successful, but they had a great disadvantage: the paddles had to be applied directly to the heart, which of course required major surgery. The first successful ‘closed chest’ defibrillation was performed by the American cardiologist Paul Maurice Zoll (1911 – 1999), of Boston in 1956, using a device with a large rechargeable battery. Zoll’s achievement was based on research in dogs and clinical research on external pacing. He later described the first time he had observed stimulation of the canine ventricle by application of electric current to the chest, “*I think I recognized at that time that a new field of electric cardiac stimulation had begun*”.

## BIPHASIC DEFIBRILLATION

The biphasic waveform was first developed by the Russian scientist Naum L Gurvich in the 1930s, after years of research in animals. It was not until the 1980s that research in the west indicated its superiority to the Lown waveform introduced by Bernard Lown in 1959. The Lown waveform was the standard for defibrillation until the late 1980s, when numerous studies showed that a biphasic truncated waveform (BTE) was better. The major advantage of biphasic over the Lown waveform was that it required less energy, which made it safer, with reduced risk of burns and muscle damage. The reduced power requirement also enabled the manufacture of lighter, more portable machines.

The BTE waveform, combined with automatic measurement of transthoracic impedance is the basis for modern defibrillators. Defibrillators have come a long way in the last half-century, and now save lives on the streets and even in the air, as well as in hospitals and ambulances. Their success in preventing deaths in cases of sudden cardiac arrest has led to the installation of defibrillation machines in airports, railway stations, restaurants, sports centers, and many other public places.

## THE ARTIFICIAL MAESTRO!

In 1882, Hugo von Ziemssen (1829 – 1902) in Germany described a case of a 42-year-old lady who had a postoperative defect in the anterior left chest wall. The heart was covered only by a thin layer of skin and was directly palpable. von Ziemssen noted that application of electrodes to the heart resulted in rhythmic stimulation only if the rate of stimulation was greater than that of the spontaneous heart rate. Slower stimulation produced erratic and sometimes slower heart rate. He also noted that the most sensitive area for stimulation was in the region of atrioventricular groove. Interestingly, this observation was made more than a decade before the description of the location of the atrioventricular node and bundle by Wilhelm His (1863 – 1934).

In 1926, the Australian anesthesiologist Mark C. Lidwill (1878 – 1968), devised a portable apparatus in which “*One pole was applied to a skin pad soaked in strong salt solution*,” while the other pole “*consisted of a needle insulated except at its point, and was plunged into the appropriate cardiac chamber. The pacemaker rate was variable from about 80 to 120 pulses per minute, and likewise the voltage was variable from 1.5 to 120 volts*”. In 1928, the apparatus was used to revive a stillborn infant whose heart continued “*to beat on its own accordt the end of 10 minutes of stimulation*”.

In 1932, the American physiologist Albert S. Hyman (1893 – 1972) described an electro-mechanical instrument of his own, powered by a spring-wound, hand-cranked motor. Hyman coined the term ‘artificial pacemaker’, in 1932, to describe this electric device that could pace the heart and lead it like an artificial maestro! The delay in publication and application of this research was attributed to the public perception of interfering with nature by ‘reviving the dead’ and ‘prolongation of life and interfering with the will of God’. In those days, death was defined as the arrest of cardiac activity and absence of pulse. Hyman did not publish data on the use of his pacemaker in humans because of adverse publicity, both among his fellow physicians, and due to newspaper reporting at the time. Lidwill may have been aware of such resistance and did not proceed with his experiments in humans.

Wilfred G. Bigelow (1913 – 2005), John A. Callaghan and John Hopps in Canada used transcutaneous electrodes to pace the right atrium in dogs. In 1949, during an experimental operation, a dog’s heart suddenly stopped. “*Out of interest and desperation*,” recalls Bigelow, “*I gave the left ventricle a good poke with a probe I was holding…All the four chambers of heart responded to it and further pokes clearly indicated that the heart was beating normally with good blood pressure*”. He discussed this event with Dr. Callaghan. Using dogs and rabbits, they studied the most effective safe electric current, and presented movies of their experiments at the Annual Surgical Congress of the American College of Surgeons, in 1950. The external pacemaker was designed and built by the Canadian electrical engineer John Hopps (1919 – 1998) based on observations by cardiothoracic surgeon Wilfred Bigelow at the Toronto General Hospital. The device was a large electrical machine with vacuum tube technology, to provide transcutaneous pacing. It was somewhat crude and painful to the patient, and being powered from an AC outlet; it carried a potential hazard of electrocution of the patient when inducing ventricular fibrillation. John Hopps himself later became a pacemaker recipient. In the cold war era, it was difficult for them to be aware of the brilliant earlier research done in the Soviet Union (USSR).

Hopps also designed the first catheter-electrode for cardiac stimulation, which was introduced via the right external jugular vein of the experimental animal. Using his external pacemaker, atrial pacing was achieved and control of the cardiac rate was accomplished. In 1950, Paul M. Zoll began work on an external pacemaker, which was used to stimulate the heart, across the chest wall. The pacemaker current put out a maximum of approximately 150 volts. Output voltage and stimulation rates were controlled from the front panel of the pacemaker. The electrodes were two, one-inch diameter metal disks placed on the right and left sides of the chest, held in place by a rubber strap and made contact via conductive electrode jelly. By 1952, the first articles concerning the pacemaker were published, and revolutionized the concept of resuscitation of the patient with heart block and asystole. External pacing was painful and required sedation. Prolonged stimulation produced local skin burns. The longest period of reported stimulation was 11 days. Zoll also later developed a bulky external transcutaneous pacemaker operated by rechargeable batteries.

## 1957: FIRST BATTERY-OPERATED PORTABLE EXTERNAL PACEMAKER

Earl Bakken (1924 –), an electrical engineer designed the first battery-operated pacemaker. Bakken and his brother-in-law Palmer Hermundslie co-founded Medtronic, in 1949, in a garage in Minneapolis. The company operated mainly as a repair service for hospital electrical equipment. At the time, Clarence Walton Lillehei (1918 – 1999), was starting a successful repair of the congenital heart defect using cross-circulation, and later on, using the heart–lung machine. About one patient in ten developed complete heart block postoperatively. Drugs such as adrenaline, atropine or isoprenaline, were helpful in the short-term, but proved disappointing over a longer period of time and could not prevent a sudden recurrence of heart block. Another solution had to be found. It was thought that temporary pacing could keep patients alive until recovery of the conducting system. The external pacing developed by Zoll was clearly inappropriate as the high voltage pacing delivered transcutaneously would be far too traumatic on these young children.

Lillehei and his co-workers developed a myocardial wire: a stainless steel wire in a Teflon sleeve. One end of this wire was implanted directly into the myocardium and the other end was exteriorized and connected to a stimulator. An indifferent electrode was buried under the skin to complete the circuit. Effective pacing needed only 1.5 volts as there was direct contact with the myocardium. There was no rejection and no damage to the beating heart and the wire could be removed easily once normal conduction resumed. The first myocardial wire was implanted in 1957, in a three-year old girl, in whom heart block had complicated the repair of Fallot’s tetralogy. Pacing was successful and the little girl soon regained sinus rhythm and survived. The myocardial wires were implanted electively in all patients; ready for immediate use should this become necessary.

Further problems soon became obvious: the stimulator was large and heavy and the system depended totally on external power supply, On October 31, 1957, a municipal power failure lasting three hours resulted in the tragic death of a baby. The day after, Lillehei requested Bakken to see if Medtronic could come up with something better. After only four weeks of experimentation in using the transistor circuit, the first battery-powered pacemaker was put in clinical use! Bakken recounts: “*Without any grandiose expectations for the device, I was moderately optimistic about what it might eventually do for Lillehei’s patients. I drove the device over to the University’s animal lab where it could be tested on a dog. Of course it worked. The next day I returned to the hospital to work on another project when I happened to walk past a recovery room and spotted one of Lillehei’s patients. The little girl was wearing the prototype I had delivered only the day before! I was stunned. I quickly tracked down Lillehei and asked him what was going on. In his typical calm, measured, no-nonsense fashion he explained that he’d been told by the lab the pacemaker worked and he didn’t want to waste another minute without it. He said he wouldn’t allow a child to die because we hadn’t used the best technology available.*” In modern terms, this was the first VOO external portable pacemaker.

## 1958: FIRST TOTALLY IMPLANTABLE INTERNAL PACEMAKER

On October 8, 1958, the first implantation of a totally internal pacemaker was performed in Sweden. The system was developed by the surgeon Ake Senning (1915 – 2000), and the physician inventor Rune Elmqvist (1906 – 1996), It was implanted on a 43-year-old engineer called Arne Larsson. This experience was reported at the Second International Conference on Medical Electronics in 1959.

Senning was the cardiac surgeon in charge of the Department of Thoracic Surgery at the Karolinska Hospital in Stockholm. He had observed Lillehei’s work with temporary external pacing. Elmqvist was a medical graduate who became an engineer. He designed a portable ECG machine and ink jet printer. These two men began to collaborate closely in 1950 and developed fibrillators and defibrillators for open heart surgery. They realized that the main problem with external pacemakers was the route for ascending infection along the wire, and planned to design a fully implantable system. Silicone transistor technology was used in the circuit. The pulse generator was powered by two rechargeable nickel-cadmium batteries. Recharging was delivered transcutaneously using external induction coils for 12 hours every week.

## THE PATIENT AND THE PROCEDURE

Arne Larsson had been hospitalized with complete heart block and frequent Stokes-Adams attacks for six months. He was having 20 to 30 attacks daily and his prognosis was poor. Treatment was maximized with ephedrine, atropine, isoprenaline, caffeine, digoxin, and whisky without success.

To avoid publicity, the implantation of the pacemaker was done in the evening when the operating rooms were empty. Two electrodes were implanted into the myocardium and tunneled to the pacemaker pulse generator placed in the abdominal wall. The first pacemaker implanted functioned only a few hours. Senning recounts: “*I implanted the first pacemaker, but it lasted only eight hours. Presumably, I had damaged the output transistor or capacitance with the catheter and I did not have the other one which was in the lab. I implanted the other one early the next morning*”. The second pacemaker functioned well for about one week. In modern terms, this was the first VOO internal pacemaker, The pulse rate was fixed at a constant rate of 70 to 80 beats per minute. The energy need was minimized, as Elmqvist used silicon transistors in the circuit. The entire unit was handmade and was encapsulated in a resin that had excellent biocompatibility. The approximate diameter was 55 mm, and thickness 16 mm.

## THE PERSONAL OUTCOME

Rune Elmqvist died in 1997, Ake Senning remained very active in the field of cardiac surgery and died in 2000. The patient Arne Larsson survived both the engineer and the surgeon who had saved his life. He required five lead systems and 22 pulse generators of 11 different models until his death from cancer on December 28, 2001, at the age of 86 years.

## 1960:CHANCE AT WORK

Wilson Greatbatch (1919), is an electrical engineer who was teaching at the University of Buffalo, where he accidentally discovered the way to make an implantable pacemaker, while working on an oscillator to aid in the recording of tachycardia.

Greatbatch describes the event this way: “*It was no accident; the Lord was working through me. The oscillator required a 10 K resistor at the transistor base. I reached into my resistor box for one, but I misread the color coding and got a 1 M resistor by mistake.*” When he plugged in the resistor, the circuit started to ‘*squeg*’ with a 1.8 millisecond pulse followed by a 1 second interval during which the transistor was cut off and drew practically no current. “*I stared at the thing in disbelief*” he said. Greatbatch realized that this small device could drive a human heart, but it wasn’t easy to find a surgeon who would believe in his idea.

William Chardack, was chief of surgery at Buffalo’s Veteran’s Hospital at the time. On May 7, 1958, Greatbatch brought what would become the world’s first implantable pacemaker to the animal laboratory at the hospital. There, Chardack and another surgeon, Andrew Gage, exposed the heart of a dog to which they touched the two pacing wires. The heart proceeded to beat in synchrony with the device. A year later, Greatbatch wrote “*I seriously doubt if anything I ever do will give me the elation I felt that day when a 2 cubic inch electronic device of my own design controlled a living heart.*” Chardack reported the first success in a human with this unit in 1960. The procedure was completed in June 1960 on a 77-year-old man, in complete heart block. Chardack implanted the lead, measured the threshold, and implanted the pulse generator. The patient survived uneventfully for two years. The system was powered by mercury–zinc battery that usually lasted for about five years.

In 1961, Chardack, Gage, and Greatbatch reported a series of 15 patients who had implanted pacemakers. Greatbatch later invented the long-life lithium–iodine battery, which powered modern pacemakers.

## DEVELOPING BETTER LEADS

Faulty batteries, body fluids leaking into the case and broken leads caused numerous pacemaker failures that required emergency operation. The main difficulty, however, was the lead. It was obvious that the myocardial wire was unsuitable for long-term use. Stimulation threshold increased after a few weeks until an exit block developed and no more capture was possible. Moreover, the wire could not resist the mechanical stresses. These technical problems contributed to the delay in the widespread use of implanted pacemakers for several years.

The technique for inserting permanent transvenous bipolar pacing electrodes was developed in 1962, by Victor Parsonnet (in USA) and by Ekstrom (in Sweden). Transvenous leads replaced myocardial leads. Pacemakers and their leads could be implanted without a thoracotomy and without general anesthesia. Lead designs were improved: ‘tined’ for passive fixation and ‘screw-in’ for active fixation.

In the early 1980s steroid-eluting leads were developed to decrease the inflammatory response evoked by the tip. Consequently, the early rise of the capture threshold was resolved and safety was enhanced.

## MORE IMPROVEMENTS

The lithium–iodine battery was developed to replace the mercury–zinc battery. This resulted in greatly increased pacemaker longevity.

The use of external pacing was reported by Zoll in the late 1950s; however, Zoll’s pacemaker was a fixed system without a demand capability and could cause ‘R on T’-induced ventricular fibrillation. At St. George’s Hospital in London, Aubrey Leatham (1920),and Geoffrey Davies (1924 – 2008) developed the first demand circuit device, which was published in 1956. However, Demand pacemakers were not in clinical use till the late sixties.

In 1972, an American-made radioisotope pacemaker was implanted by Parsonnet. These nuclear pacemakers had an expected life of 20 years, but went out of fashion mainly due to the need for extensive regulatory paperwork.

Radio-frequency telemetry made pacemakers non-invasively programmable in the mid-70s. Most pacing parameters could be remotely adjusted to follow the changing clinical needs of the patient. Modern telemetry allows follow-up of pacemaker patients anywhere in the world, by the use of telephone lines and mobile phones. Modern pacemakers can store some medical information about the patient, record cardiac events, and ECG, By the end of the ‘70s dual-chamber pacemakers were developed to pace and sense in both the atria and ventricles. Synchronized DDD timing made it possible to preserve the atrial contribution to ventricular filling as well as to track the intrinsic atrial rate. In the mid-1980s, rate-responsive pacemakers were designed.

## BIVENTRICULAR PACING

Biventricular pacing for heart failure was introduced and started the revolution of Cardiac Resynchronization Therapy (CRT). An additional lead was introduced via the coronary sinus toward the left ventricle. The right ventricle (via the standard lead) and the left ventricle (via coronary sinus lead) were paced simultaneously to resynchronize their contraction and improve the symptoms and survival of properly selected heart failure patients.

## INTERNAL DEFIBRILLATORS

The development of the Internal defibrillators (ICD) was pioneered at the Sinai Hospital in Baltimore by a team that included Michel Mirowski (1924 – 1990) and Morton Mower (1933). They started their research in 1969, but it took 11 years before they treated their first patient. More than a decade of research went into the development of an implantable defibrillator that would automatically sense the onset of ventricular fibrillation and deliver an electric counter-shock converting the heart to sinus rhythm.

Leading experts in the field of arrhythmias and sudden death were skeptical. There was doubt that the idea of an implantable defibrillator would ever become a clinical reality. In 1972, Bernard Lown, the inventor of the Lown waveform, which was the standard for defibrillation until the late 1980s, stated: “*The very rare patient who has frequent bouts of ventricular fibrillation is best treated in a Coronary Care Unit and is better served by an effective antiarrhythmic program or surgical correction of inadequate coronary blood flow or ventricular malfunction. In fact, the implanted defibrillator system represents an imperfect solution in search of a plausible and practical application*”. Just eight years later, the first ICD device was implanted in February 1980, at Johns Hopkins Hospital by Levi Watkins. The patient survived for 10 years and died from unrelated causes.

Initially, ICDs were implanted via surgical thoracotomy with the defibrillator patches applied directly to the epicardium or pericardium. The leads were attached to the device, which was placed in a large subcutaneous abdominal wall pocket. Most ICDs nowadays, are implanted transvenously with the devices placed in the left pectoral region similar to pacemakers, and can deliver CRT pacing, cardioversion, and defibrillation.

## THE ROLE OF SURGEONS AND CARDIOLOGISTS IN DEVELOPING PACEMAKERS: TEAM WORK SUCCESS STORY

Cardiothoracic surgeons were leading pioneers in developing and implementing the clinical use of cardiac pacing and defibrillation. Great pioneers, such as Beck, Bigelow, Lillehei, Senning, Chardack, Parsonnet, Watkins, and others, were leaders in this field. Almost all early clinical applications of cardiac pacing and defibrillation required an operative approach, before further technical advancement allowed transvenous placement under local anesthesia. Young cardiologists and cardiothoracic surgeons may be surprised to know the significant contribution of pioneer surgeons to understanding and treating cardiac arrhythmias. I recall a time when I was a resident in cardiothoracic surgery at the Medical University of South Carolina in 1981, when we used to do all transvenous pacemaker implantation, and started the early clinical trials of dual chamber (DDD) pacemakers. Cardiology fellows had just started to join us then to learn these techniques. Yet, the story of cardiac pacing is a story of success in cooperation in this magnificent century. The collaboration between engineers, cardiologists, cardiothoracic surgeons, and patients was the fundamental driving force for the development of new hope for cardiac patients, and the growth of a significant global industry. The cooperation between these pioneers was a success story of team work; doctors working together, and extending caring hands to help their patients.
